# Supplementary material for: Macrophage-augmented intestinal organoids model virus-host interactions in enteric viral diseases and facilitate therapeutic development
Source: Nat Commun. 2025 May 14;16:4475. doi: 10.1038/s41467-025-59639-9 (PMC12078800; doi:10.1038/s41467-025-59639-9)
Supplement: Supplementary file 1 — Supplementary Information [file 41467_2025_59639_MOESM1_ESM.pdf]

Supplementary information to

Macrophage-augmented intestinal organoids model virus-host interactions in enteric viral diseases and facilitate therapeutic development

Guige Xu et al.

Supplementary Figure 1

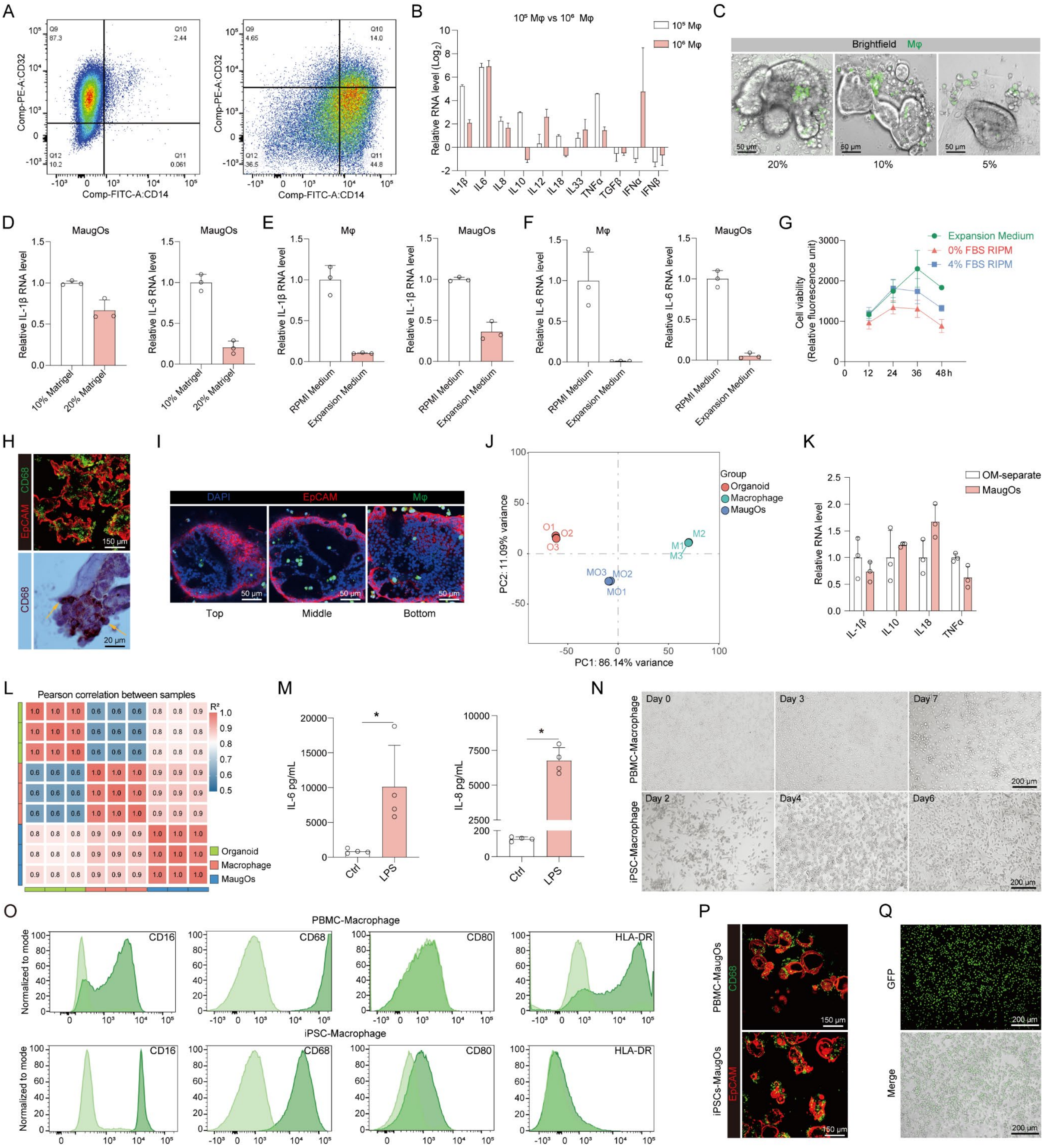

Supplementary Figure 1. Establishment and characterization of macrophage-augmented intestinal organoids.

(A) FACS characterization of macrophages differentiated from THP-1 monocytes by measuring the expression of representative immune markers (CD32, CD14). The left image was for THP-1 monocytes and the right image was for THP-1 monocyte-differentiated macrophages.

(B) The expression of inflammatory genes in MaugOs incorporated with the number of 10^5 or 10^6 THP-1 derived macrophages upon stimulation of 1 μg/mL LPS for 12 hours. Each group was compared to its respective control without LPS treatment (n = 3).

(C) Morphological observation of MaugOs cultured in different concentrations of Matrigel.

(D) QRT-PCR quantification of IL-1β and IL-6 gene expression in LPS treated MaugOs cultured with different concentrations of Matrigel (n = 3).

(E to F) QRT-PCR quantification of IL-1β (E) and IL-6 (F) gene expression in LPS treated THP-1 macrophages and MaugOs using different culture medium (n = 3).

(G) Cell viability of MaugOs cultured in different medium for two days (n = 3). Relative fluorescence units were obtained at an excitation wavelength of 530 nm and an emission wavelength of 590 nm (λExc 530nm/λEm 590nm).

(H) Immunofluorescence and immunohistochemistry (IHC) staining of MaugOs integrated THP-1 monocytes-derived macrophages. Epithelial membrane of organoids was stained by EpCAM (red); THP-1 monocytes-derived macrophages stained by CD68 (green) in Immunofluorescence panel. Yellow arrow indicates the macrophages of MaugOs stained by CD68 in IHC panel. Scale bar, 150 µm and 20 µm.

(I) Representative immunofluorescence staining images of MaugOs at different scanning layers. These images were related to MaugOs in Figure 1D. Scale bar, 50 µm.

(J) Principal component analysis for genome-wide RNA sequencing of organoids, THP-1 macrophages and MaugOs (n = 3).

(K) Quantification of IL-1β, IL-10, IL-18, and TNFα gene expression in MaugOs compared to the expression in separately cultured organoids and THP-1 macrophages (n = 3). Note: For separately cultured organoids and macrophages, the lysed organoids and macrophages were combined for RNA isolation.

(L) Correlation matrix of transcriptomic profiles of macrophages, organoids and MaugOs (n = 3). A value of 1 represents complete correlation, and a value of 0 represents no significant correlation.

(M) Quantification of IL-6 and IL-8 protein production by ELISA in MaugOs integrated THP-1 monocytes-derived macrophages (n = 4).

(N) Bright field images of PBMC monocytes-derived and iPSCs-derived macrophages during the differentiation process. Scale bar, 200 µm.

(O) FACS characterization of macrophages differentiated from PBMC- or iPSCs-monocytes by measuring the expression of representative immune markers (CD16, CD68, CD80 and HLA-DR).

(P) Immunofluorescence staining images of MaugOs integrated PBMC monocytes-derived macrophages and iPSCs-derived macrophages. Epithelial membrane of organoids was stained by EpCAM (red); PBMC monocytes-derived macrophages and iPSCs-derived macrophages stained by CD68 (green). Scale bar, 150 µm.

(Q) Morphological observation of iPSCs-derived macrophages (Macrophages stably express GFP, green color). Scale bar, 200 µm.

Data were presented as means of biological replicates ± SEM. Statistical analysis was performed using the two-tailed Mann–Whitney test. *$p < 0.05$.

Supplementary Figure 2

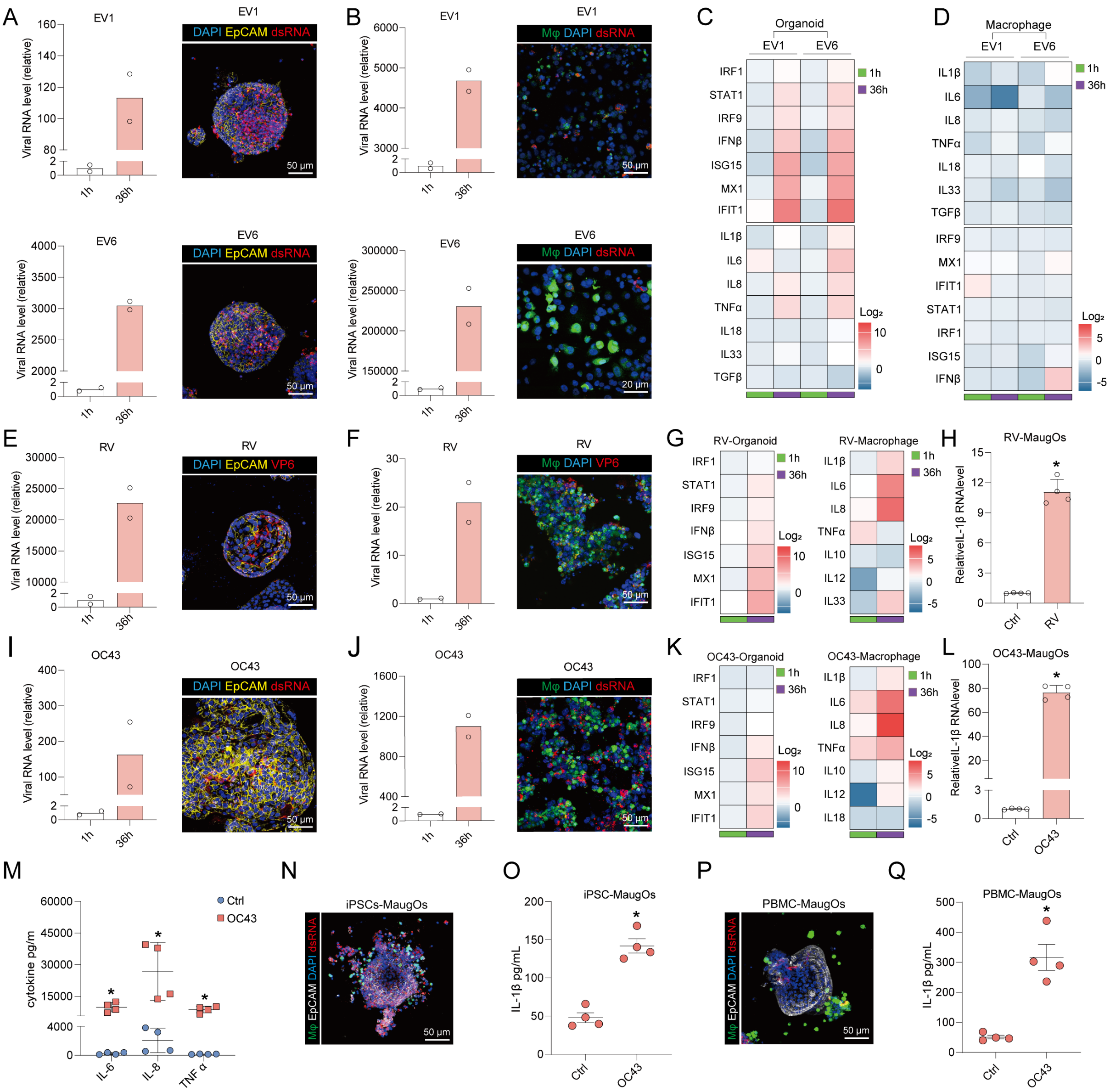

Supplementary Figure 2. MaugOs recapitulate enteric viral infections and inflammatory response.

(A) QRT-PCR quantification of viral RNA level (n = 2), and confocal imaging of viral replicating dsRNA (red) in intestinal organoids infected with EV1 and EV6 at 36 hours post-infection. The epithelial membrane of organoids was stained by EpCAM (yellow).

(B) QRT-PCR quantification of viral RNA level (n = 2), and confocal imaging of viral replicating dsRNA (red) in THP-1 monocytes differentiated macrophages infected with EV1 and EV6. THP-1 macrophages were stained with CFSE (green).

(C-D) QRT-PCR quantification of inflammatory and antiviral associated genes expression in organoids (C) and THP-1 macrophages (D) upon the infection of EV1 and EV6 (n = 2-3).

(E) QRT-PCR quantification of rotavirus RNA level (n = 2) and confocal imaging of rotavirus VP6 protein (red) in intestinal organoids at 36 hours after rotavirus infection. Epithelial membrane of organoids was stained with EpCAM (yellow). Scale bar, 50 µm.

(F) QRT-PCR quantification of rotavirus RNA level (n = 2) and confocal imaging of rotavirus VP6 protein (red) in THP-1 macrophages at 36 hours after rotavirus infection. Scale bar, 50 µm.

(G) QRT-PCR quantifying the expression of antiviral ISGs in organoids and inflammatory-associated genes in THP-1 macrophages triggered by rotavirus infection (n = 2-3).

(H) QRT-PCR quantification of IL-1β gene expression in MaugOs at 36 hours after rotavirus infection (n = 4).

(I) QRT-PCR quantification of OC43 RNA level (n=2) and confocal imaging of viral replicating dsRNA (red) in intestinal organoids at 36 hours after OC43 virus inoculation. Epithelial membrane of organoids was stained with EpCAM (yellow). Scale bar, 50 µm.

(J) QRT-PCR quantification of viral RNA level (n = 2) and confocal imaging of viral replicating dsRNA (red) in THP-1 macrophages infected with OC43 virus at 36 hours post-inoculation. THP1-derived macrophages were labeled by CFSE (green). Scale bar, 50 µm.

(K) QRT-PCR quantification of antiviral ISGs expression in organoids and inflammatory-associated genes expression in THP-1 macrophages upon OC43 infection (n = 2-3).

(L) Quantification of IL-1β gene expression in MaugOs at 36 hours after OC43 infections compared to uninfected MaugOs (n = 4).

(M) ELISA quantification of IL-6, IL-8 and TNF-α that secreted into supernatant of uninfected and OC43 infected MaugOs (n = 4).

(N) Confocal imaging of OC43 virus infection in MaugOs integrated iPSCs-derived macrophages. Scale bar, 50 µm.

(O) Quantification of IL-1β production in uninfected and OC43 infected MaugOs integrated iPSCs-derived macrophages (n = 4).

(P) Confocal imaging of OC43 virus infection in MaugOs integrated PBMC-derived macrophages. Scale bar, 50 µm.

(Q) Quantification of IL-1β production in uninfected and OC43 infected MaugOs integrated PBMC-derived macrophages (n = 4).

Data were presented as means of biological replicates ± SEM. Statistical analysis was performed using the two-tailed Mann–Whitney test. *$p < 0.05$.

Supplementary Figure 3

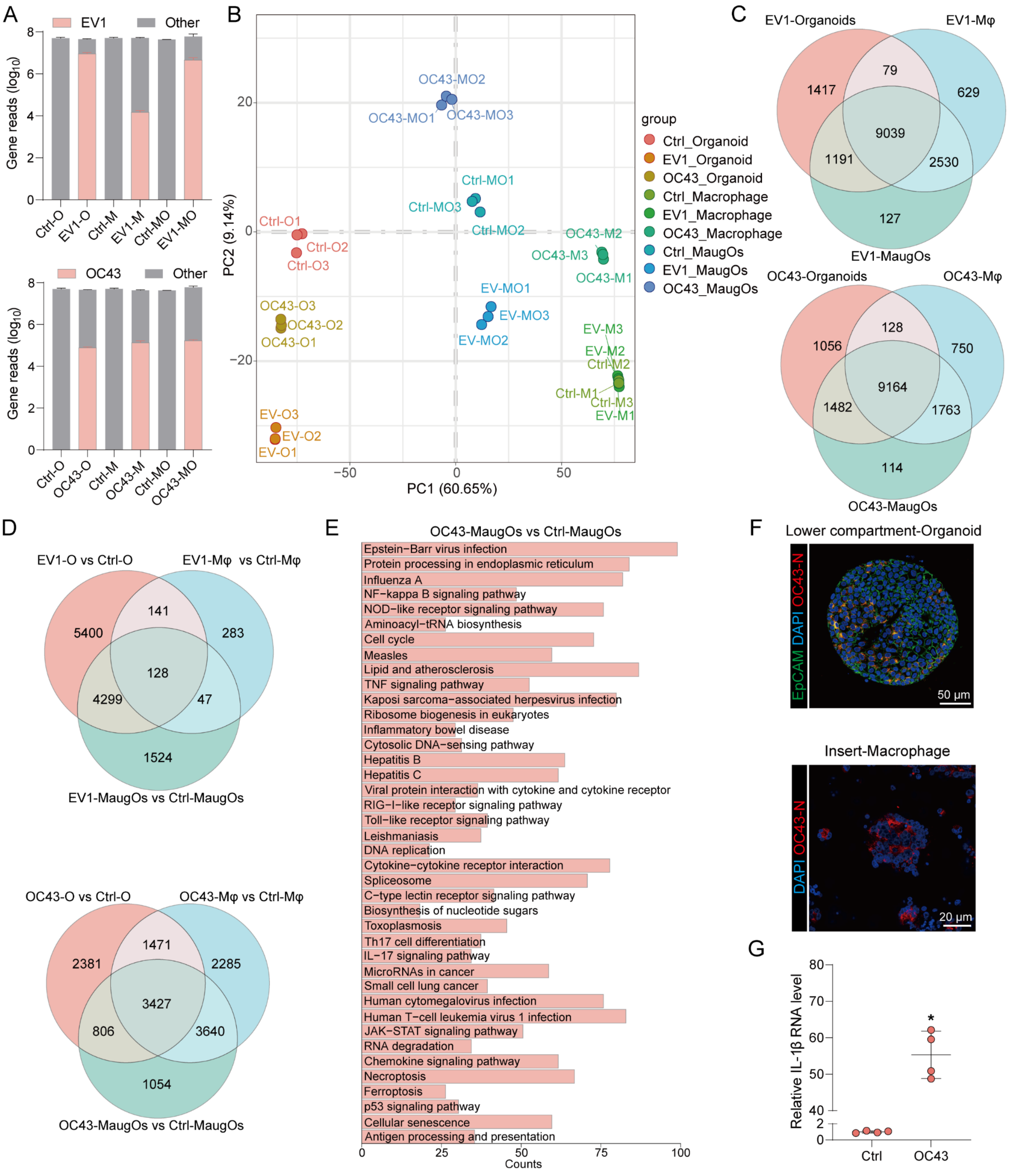

Supplementary Figure 3. Coronavirus OC43 infection in MaugOs triggers both antiviral and inflammatory responses

(A) The reads of mapped OC43 or EV1 viral transcripts in organoids, THP-1 macrophages and MaugOs integrated THP-1 macrophages (n = 3).

(B) Principal component analysis of infected and uninfected organoids, THP-1 macrophages and MaugOs integrated THP-1 macrophages (n=3).

(C) Venn diagram of overlapped differentially expressed genes in macrophages (Mφ), intestinal organoids and MaugOs upon EV1 or OC43 infection at 36 hour post-inoculation.

(D) Venn diagram of overlapped differentially expressed genes among EV1- or OC43-infected macrophages (Mφ), intestinal organoids and MaugOs compared to uninfected sample at 36 hour post-inoculation (n=3).

(E) Top 40 significantly regulated KEGG pathways in OC43-infected MaugOs compared to MaugOs without OC43 infection (n = 3).

(F) Confocal imaging of OC43-N protein (red) in organoids at the lower compartment and macrophages at the upper compartment, with the epithelial membrane of organoids stained with EpCAM (green). Scale bar, 50 μm and 20 μm.

(G) QRT-PCR quantification of IL-1β gene expression in apical THP-1 macrophages (n = 4).

Data were presented as means of biological replicates ± SEM. Statistical analysis was performed using the two-tailed Mann–Whitney test. *$p < 0.05$.

Supplementary Figure 4

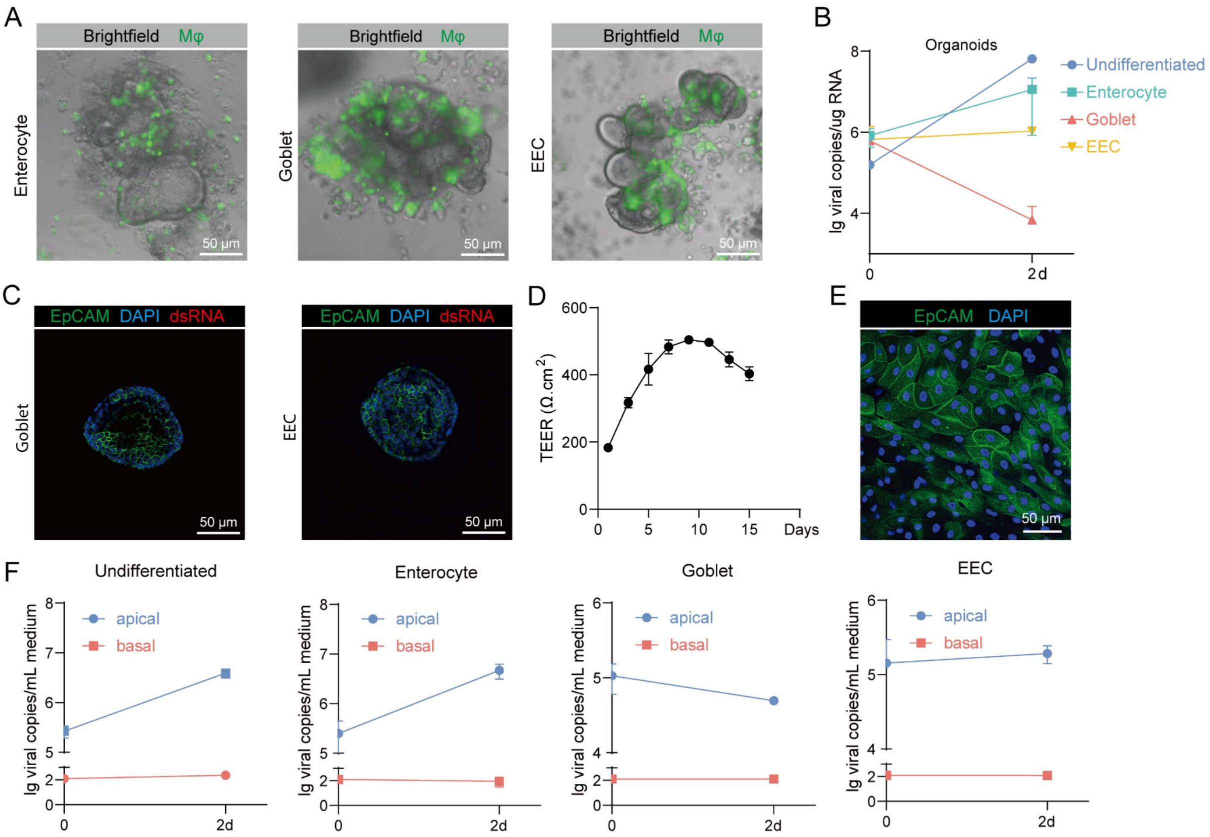

Supplementary Figure 4. Establishment of MaugOs using differentiated intestinal organoids.

(A) Morphology of MaugOs integrated with enterocytes-, goblet cells- and enteroendocrine cells -differentiated organoids. THP-1 -derived macrophages were stained by CFSE (green). Scale bar, 50 µm.

(B) Quantification of OC43 viral RNA level in undifferentiated and differentiated organoids at 48 hours post infection (n = 3).

(C) Confocal imaging of viral replicating dsRNA in goblet cell-differentiated organoids and enteroendocrine cells (EEC)-differentiated organoids at 48 hours post infection (dsRNA fluorescence signal was not detected). Scale bar, 50 µm.

(D) TEER values of the monolayer in the trans-well membrane test at 1, 3, 5, 7, 9, 11, 13, and 15 days after seeding the cells.

(E) Confocal imaging of trans-well monolayer showing the epithelial membrane of organoid cells stained with EpCAM (green). Scale bar, 50 µm.

(F) Quantification of OC43 viral RNA levels in the culture medium of apical and basolateral compartment in trans-well system at 48 hours post-infection (n = 3).

Supplementary Figure 5

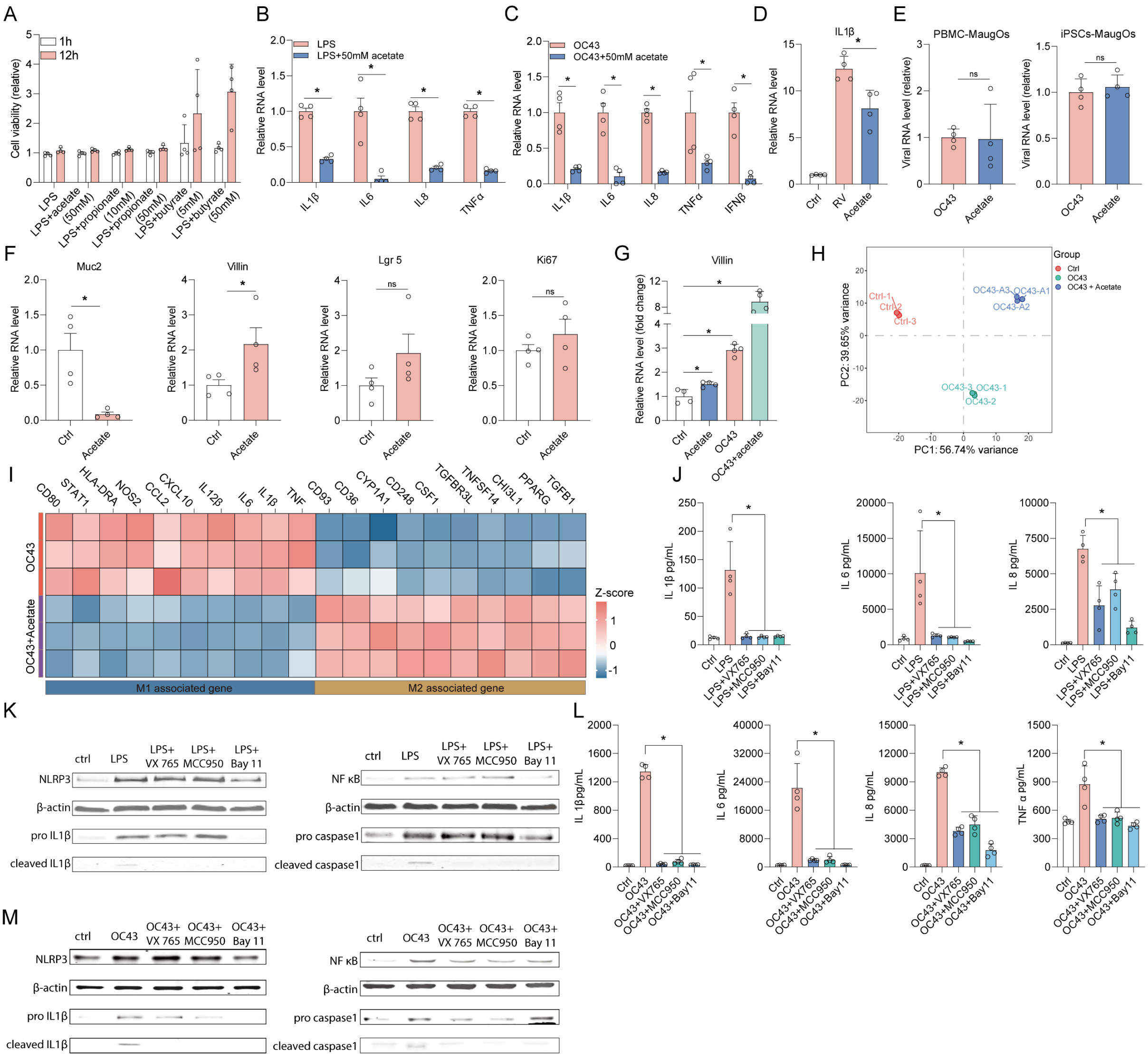

Supplementary Figure 5. Characterizing the function of acetate on enteric viral infection and inflammatory response.

(A) Cell viability of MaugOs after treatment with LPS and various SCFAs for 12 hours (n = 4).

(B) The effect of acetate on the expression of IL-1β, IL6, IL8, and TNFα in LPS-treated MaugOs quantified by qRT-PCR (n = 4).

(C) The effect of acetate on the expression of IL-1β, IL6, IL8, TNFα, and IFNβ in OC43-infected MaugOs quantified by qRT-PCR (n = 4).

(D) The effect of acetate on the expression of IL-1β in rotavirus-infected MaugOs quantified by qRT-PCR (n = 4).

(E) Quantification of viral RNA level in OC43-infected MaugOs integrated PBMC- or iPSCs derived macrophages with or without 50mM acetate treatment for 36 hours (n = 4).

(F) The effect of acetate on the expression of Villin, Muc2, Lgr5, and Ki67 in organoids quantified by qRT-PCR (n = 4).

(G) Quantification of IL-1β gene expression in MaugOs integrated THP-1 derived macrophages. MaugOs were triggered by OC43 infection with or without 50mM acetate treatment for 36 hours (n = 4).

(H) PCA of different MaugOs groups with three replicates per group (control: uninfected MaugOs; OC43: OC43 infected MaugOs; OC43+Acetate: 50 mM acetate treatment in OC43-infected MaugOs).

(I) Profiling the influence of acetate on representative gene markers expression of M1- and M2-phenotype macrophage. OC43: OC43 virus infected-MaugOs; Acetate: OC43 virus infected-MaugOs with 50 mM acetate treatment for 36 hours (n = 3).

(J) Quantification of the IL-1β protein production in the supernatant of MaugOs integrated THP-1 derived macrophages. MaugOs were triggered by LPS with or without treatment of VX765, MCC950 and BAY 11 for 36 hours (n = 4).

(K) The inhibitory effect of VX765, MCC950 and BAY 11 treatment on the protein level of NF-κB and NLRP3 signaling cascade in LPS treated MaugOs at 36 hours determined by western blotting. NF-κB, NLRP3, pro IL-1β and pro caspase 1 were detected from cell lysates; Cleaved caspase-1 and cleaved IL-1β were detected from culture supernatant.

(L) Quantification of the IL-1β protein production in the supernatant of MaugOs integrated THP-1 derived macrophages. MaugOs were triggered by OC43 infection with or without treatment of VX765, MCC950 and BAY 11 for 36 hours (n=4).

(M) The inhibitory effect of VX765, MCC950 and BAY 11 treatment on the protein level of NF-κB and NLRP3 signaling cascade in OC43-infected MaugOs at 36 hours. NF-κB, NLRP3, pro IL-1β and pro caspase 1 were detected from cell lysates; Cleaved caspase-1 and cleaved IL-1β were detected from culture supernatant.

Data were presented as means of biological replicates ± SEM. Statistical analysis was performed using the two-tailed Mann–Whitney test. *$p < 0.05$.

Supplementary Figure 6

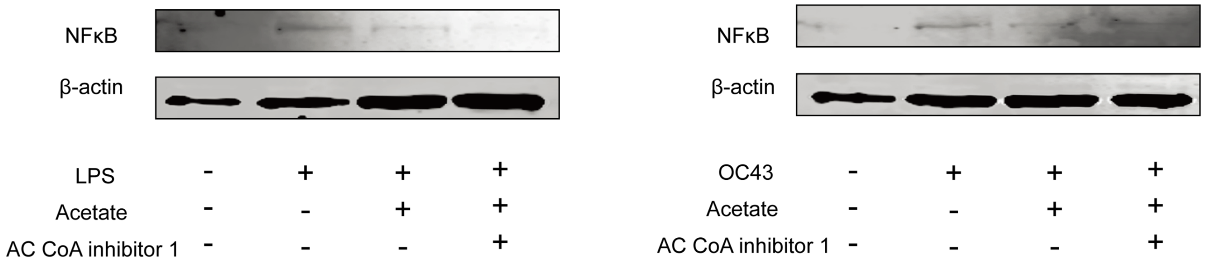

Supplementary Figure 6. Dissecting the mechanism-of-action of the anti-inflammatory effect of acetate. The effect of AC-CoA inhibitor 1 on the protein level of NF-κB signaling pathway in LPS-treated or OC43-infected MaugOs at 36 hours determined by western blotting.

Supplementary Figure 7

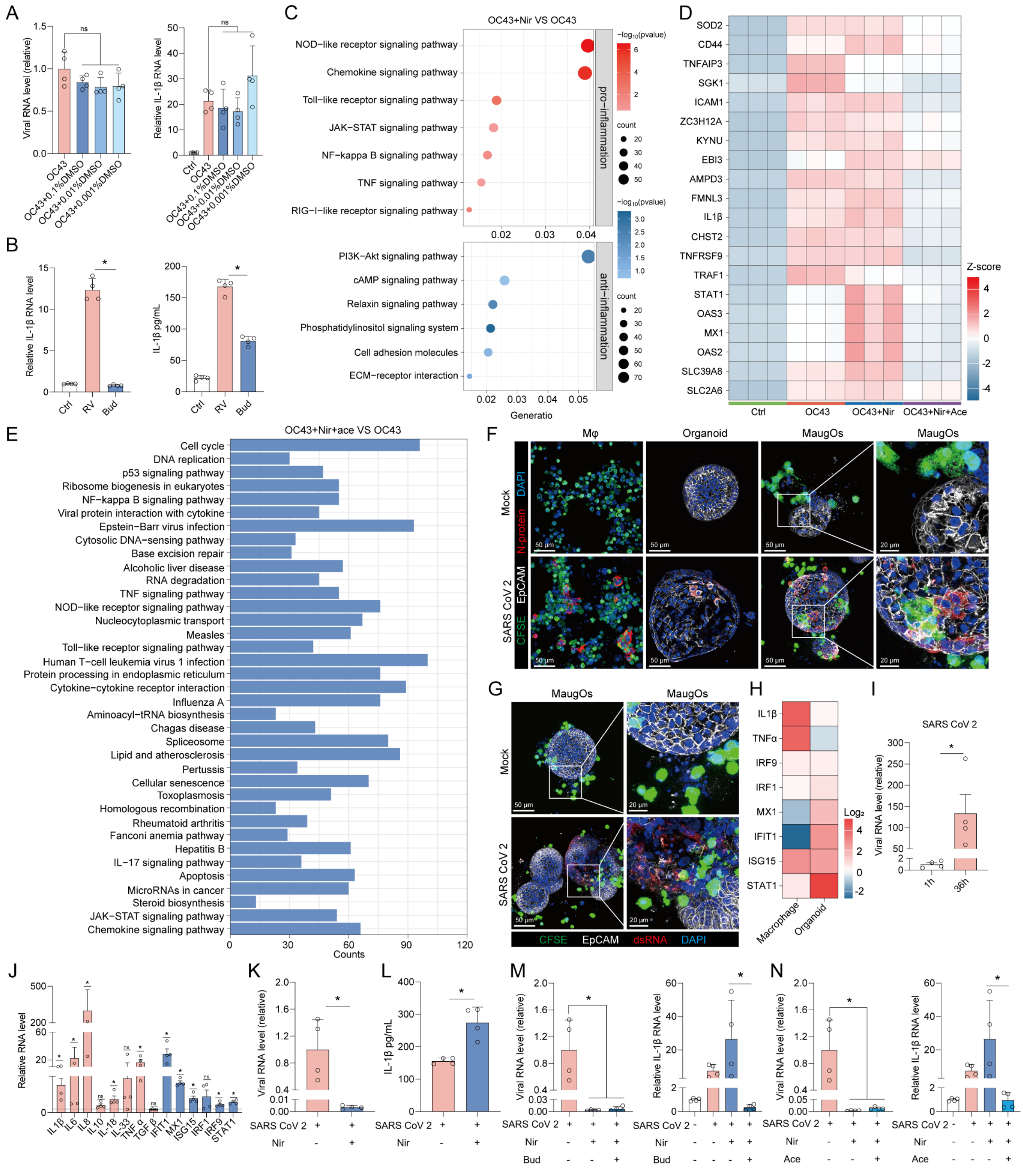

Supplementary Figure 7. Devising combination treatment against enteric viral infections in MaugOs.

(A) Quantification of OC43 viral RNA and IL-1β gene expression in OC43 infected MaugOs after treatment with varying concentrations of DMSO for 36 hours (n = 4).

(B) Quantification of IL-1β gene expression and protein production in rotavirus-infected MaugOs after budesonide treatment for 36 hours (n = 4).

(C) Significantly regulated pathways by KEGG analysis at 36 hours in OC43 infected MaugOs with 1 μM nirmatrelvir treatment ($p < 0.05$), compared with the non-treatment group. Red: upregulated; blue: downregulated (n = 3).

(D) Top 20 significantly regulated genes upon OC43 infection in MaugOs (n = 3).

(E) Top 40 significantly regulated KEGG pathways in OC43-infected MaugOs compared to MaugOs without OC43 infection (n = 3).

(F) Immunofluorescence staining of SARS-CoV-2 N-protein (red) in THP-1 macrophages, organoids, and MaugOs at 36 hours post virus inoculation. Macrophages were marked by CFSE (green); Organoid cell membrane was stained by EpCAM (white); Cell nucleus (DAPI, blue). Scale bar, 50 µm.

(G) Immunofluorescence staining of SARS-CoV-2 dsRNA (red) in MaugOs at 36 hours post virus inoculation. Macrophages were marked by CFSE (green); Organoid cell membrane was stained by EpCAM (white); Cell nucleus (DAPI, blue). Scale bar, 50 µm.

(H) The expression of inflammatory and interferon associated genes triggered by SARS-CoV-2 infection in macrophage and organoids at 36 hours quantified by qRT-PCR (n = 2).

(I) QRT-PCR quantification of viral RNA level in SARS-CoV-2 infected MaugOs at 1 hour and 36 hour post-inoculation (n = 4).

(J) The expression of inflammatory and interferon associated genes triggered by SARS-CoV-2 infection in MaugOs at 36 hours quantified by qRT-PCR (n = 4).

(K) The effect of 1µM nirmatrelvir treatment on SARS-CoV-2 viral RNA level in MaugOs at 36 hours post-treatment (n = 4).

(L) The production of IL-1β in the supernatant of SARS-CoV-2 infected MaugOs after treatment of 1 µM nirmatrelvir for 36 hours (n = 4).

(M) Quantification of SARS-CoV2 viral RNA and IL-1β gene expression in MaugOs after combination treatment of 1 µM nirmatrelvir and 1 µM budesonide for 36 hours (n = 4).

(N) Quantification of SARS-CoV2 viral RNA and IL-1β gene expression in MaugOs after combination treatment of 1 µM nirmatrelvir and 50 mM acetate for 36 hours (n = 4).

Data were presented as means of biological replicates ± SEM. Statistical analysis was performed using the two-tailed Mann–Whitney test. *$p < 0.05$.

Supplementary Table 1. Detailed information of genes expressed exclusively in MaugOs, related to Fig. 1E.

Gene name	Gene biotype
CENPS	protein_coding
RF00019	misc_RNA
MIR6730	miRNA
AL033527.3	antisense
AC093423.2	sense_intronic
MFSD14A	protein_coding
AL451085.2	antisense
SNRPGP10	processed_pseudogene
AC138393.3	lincRNA
AL512343.2	antisense
RNU4-21P	snRNA
AL355472.1	antisense
SOS1-IT1	sense_intronic
AC062037.2	antisense
FAHD2B	protein_coding
RF00019	misc_RNA
RF02271	misc_RNA
MIR6512	miRNA
SNORA75	snoRNA
LINC02585	antisense
AC137630.3	antisense
AC112484.4	TEC
RNU6-720P	snRNA
AC104411.1	antisense
RNU7-82P	snRNA
RNU6-315P	snRNA
RF00019	misc_RNA
RF00554	snoRNA
AC079921.2	lincRNA
AC093827.4	antisense
SHLD3	protein_coding
AC010359.1	lincRNA
GUSBP9	unprocessed_pseudogene
AC116347.1	processed_pseudogene
RNU6-444P	snRNA
AL023693.1	antisense
SNORA15B-1	snoRNA
MIR5692C2	miRNA
AC018638.3	processed_pseudogene
RNA5SP243	rRNA_pseudogene
MIR503	miRNA
IKBKGP1	unprocessed_pseudogene
F8A2	protein_coding
AC067838.1	lincRNA
RNU6-323P	snRNA
RF00019	misc_RNA
RF00019	misc_RNA
MIR181B2	miRNA

RNU4-39P	snRNA
DEFB131B	protein_coding
RF00409	snoRNA
RF00019	misc_RNA
RPL11P3	processed_pseudogene
MIR4685	miRNA
MIR4295	miRNA
AC005342.2	antisense
AC048341.2	lincRNA
AC084032.1	lincRNA
RF00019	misc_RNA
RNU2-7P	snRNA
RN7SL49P	misc_RNA
SNORA79B	snoRNA
RNU6-341P	snRNA
RNU6-689P	snRNA
AC135983.5	processed_pseudogene
ZNF444P1	processed_pseudogene
RF00093	snoRNA
AC009065.4	lincRNA
AC108134.4	sense_overlapping
NPIPA7	protein_coding
MIR3181	miRNA
GPS2	protein_coding
MIR1180	miRNA
MIR6779	miRNA
RF00019	misc_RNA
SNORD1B	snoRNA
AC087222.1	lincRNA
SNRPGP2	processed_pseudogene
AL031665.2	antisense
AL390198.2	unprocessed_pseudogene
MIR4755	miRNA
SNORA60	snoRNA
AL121845.4	antisense
IZUMO4	protein_coding
AC011447.4	unprocessed_pseudogene
AD001527.1	antisense
RF00019	misc_RNA
MIR6796	miRNA
ZNF836	protein_coding
PSMA6P1	processed_pseudogene
AC245060.2	lincRNA
AL021878.2	sense_intronic

Supplementary Table 2. Detailed information of genes expressed exclusively in EV1 infected MaugOs, related to Supplementary Fig. 3C.

Gene name	Gene biotype
RPL23AP24	processed_pseudogene
MMP23A	unprocessed_pseudogene
ZBTB40-IT1	sense_intronic
AL033527.2	antisense
RNU5F-1	snRNA
AL356968.2	processed_pseudogene
MIR6500	miRNA
RNU6-877P	snRNA
DLSTP1	processed_pseudogene
AL139421.1	processed_pseudogene
RNU6-817P	snRNA
AC245100.5	processed_pseudogene
MIR4258	miRNA
AL355472.1	antisense
CHAC2	protein_coding
MIR5192	miRNA
MIR5696	miRNA
AC108463.1	processed_pseudogene
AC104653.2	lincRNA
RNU6-377P	snRNA
RF00019	misc_RNA
RNU4-78P	snRNA
C3orf18	protein_coding
AC026877.1	processed_pseudogene
LINC02035	lincRNA
RF00019	misc_RNA
RF01210	snoRNA
RF01241	snoRNA
HTRA3	protein_coding
RF00026	snRNA
SNORA26	snoRNA
AC018797.2	antisense
RNU6-540P	snRNA
AC139495.1	transcribed_unprocessed_pseudogene
AC139272.1	unprocessed_pseudogene
AC008438.1	antisense
SNORA38	snoRNA
ABHD16A	protein_coding
RF00019	misc_RNA
RNU6-444P	snRNA
MIR1273C	miRNA
MIR3146	miRNA
AC005154.1	processed_pseudogene
SNORA5A	snoRNA
VN1R42P	processed_pseudogene
MIR4284	miRNA
SDHAF3	protein_coding
AC004975.1	processed_pseudogene

GK-AS1	antisense
SNORA11	snoRNA
ETDC	protein_coding
MIR3149	miRNA
FAM92A	protein_coding
RNU6-875P	snRNA
MIR7848	miRNA
RNA5SP530	rRNA
RF00019	misc_RNA
RNU6-1160P	snRNA
RF00019	misc_RNA
RNU6-878P	snRNA
AP006296.1	processed_pseudogene
IMMP1L	protein_coding
RNU6-45P	snRNA
MIR6750	miRNA
AP001893.1	antisense
AC022400.1	antisense
RNU6-780P	snRNA
RNU6-422P	snRNA
AL451069.1	lincRNA
RF00019	misc_RNA
FAM86FP	transcribed_unprocessed_pseudogene
AC009318.2	antisense
GPD1	protein_coding
RNU7-4P	snRNA
THAP2	protein_coding
AL157932.1	antisense
AL157813.1	sense_intronic
BIVM	protein_coding
AL132800.1	antisense
SNORD127	snoRNA
MIR4505	miRNA
RAP1AP	processed_pseudogene
LINC02328	lincRNA
RF00019	misc_RNA
AL138976.2	antisense
AC060814.1	processed_pseudogene
EIF3J-DT	lincRNA
RF00019	misc_RNA
AC100830.2	antisense
AC064799.1	processed_pseudogene
RPS17	protein_coding
RNU6-1111P	snRNA
AC027020.2	lincRNA
AL031716.1	antisense
GLIS2-AS1	lincRNA
AC126755.2	protein_coding
SNORA46	snoRNA
MIR1972-2	miRNA
SNORD71	snoRNA
RF00019	misc_RNA

RF00598	snoRNA
MIR6774	miRNA
AC015922.1	unprocessed_pseudogene
AC090615.1	unprocessed_pseudogene
AC104564.3	sense_intronic
AC138207.3	processed_pseudogene
TBC1D3G	protein_coding
MIR4734	miRNA
RF00422	scaRNA
AC111186.1	processed_pseudogene
AC087222.1	lincRNA
AC090772.4	TEC
RF00275	snoRNA
RPL37AP1	processed_pseudogene
MIR6812	miRNA
AC008752.3	unprocessed_pseudogene
AC005546.1	lincRNA
MIR3188	miRNA
RF00611	snoRNA
AC002398.1	antisense
AC006213.5	sense_intronic
MIR4324	miRNA
AP000526.1	sense_intronic
RNA5SP494	rRNA_pseudogene
AL031186.1	antisense
AL031593.1	lincRNA
AC007325.4	protein_coding

Supplementary Table 3. Detailed information of genes expressed exclusively in OC43 infected MaugOs, related to Fig. 3C.

Gene name	Gene biotype
RF00019	misc_RNA
UQCRHL	protein_coding
OSCP1	protein_coding
CYP4X1	protein_coding
RAB3B	protein_coding
AL049597.2	antisense
OVGP1	protein_coding
RNY1P13	misc_RNA
HMGCS2	protein_coding
RF00015	snRNA
S100A2	protein_coding
AL353807.4	processed_pseudogene
MFSD4A	protein_coding
CAPN9	protein_coding
RPL35P1	processed_pseudogene
RNU6-137P	snRNA
AC016735.1	lincRNA
AC007283.2	processed_pseudogene
ALPP	protein_coding
RF00019	misc_RNA
MIR191	miRNA
CHCHD6	protein_coding
AC084036.1	antisense
AC080013.6	lincRNA
UBE2V1P2	processed_pseudogene
RN7SL738P	misc_RNA
AC144530.1	processed_pseudogene
RNA5SP155	rRNA_pseudogene
RPS29P11	processed_pseudogene
SNORA26	snoRNA
RF00019	misc_RNA
SEMA5A	protein_coding
AC006077.2	TEC
AL139095.2	processed_pseudogene
AL031775.1	antisense
MUCL3	protein_coding
AL603910.1	antisense
MIR3662	miRNA
SLC22A3	protein_coding
MIR339	miRNA
IGFBP1	protein_coding
RNU6-1091P	snRNA
PEG10	protein_coding
MIR93	miRNA
RNA5SP243	rRNA_pseudogene
FAM131B	protein_coding
AC005586.1	antisense
MIR6089	miRNA

REPS2	protein_coding
NHS	protein_coding
SLC9A7	protein_coding
AL139396.1	processed_pseudogene
RPS23P8	processed_pseudogene
ZNF185	protein_coding
AC144568.1	processed_pseudogene
AC016065.1	antisense
RNU6-892P	snRNA
RNU7-181P	snRNA
RN7SL5P	misc_RNA
SLC4A1APP1	processed_pseudogene
GNA14	protein_coding
RF00019	misc_RNA
STXBP1	protein_coding
RNU7-171P	snRNA
AL391056.1	lincRNA
PRRT1B	protein_coding
MUC5AC	protein_coding
AC104563.1	processed_pseudogene
AP003555.3	lincRNA
PLEKHB1	protein_coding
LIPT2	protein_coding
AP002360.1	lincRNA
RF00019	misc_RNA
KAT6B	protein_coding
EIF5AL1	protein_coding
HABP2	protein_coding
PTMAP4	processed_pseudogene
EIF2S3B	protein_coding
SNORA2A	snoRNA
KRT6B	protein_coding
AC073896.3	antisense
MIRLET7I	miRNA
AC069234.5	lincRNA
AL161719.1	lincRNA
MYO16-AS1	antisense
RN7SL3	misc_RNA
RHOV	protein_coding
AC044787.1	processed_pseudogene
RNU6-807P	snRNA
NPW	protein_coding
CLUAP1	protein_coding
MIR6769A	miRNA
UBE2MP1	processed_pseudogene
CCDC102A	protein_coding
TPPP3	protein_coding
RF00019	misc_RNA
IL17C	protein_coding
AC138207.3	processed_pseudogene
TBC1D3G	protein_coding
ANKFN1	protein_coding

SNORD1B	snoRNA
AL035071.1	lincRNA
RPL37AP1	processed_pseudogene
SNORD12C	snoRNA
EEF1A2	protein_coding
AC005256.1	lincRNA
ZNF561-AS1	processed_transcript
RF00285	snoRNA
APOC1P1	transcribed_unprocessed_pseudogene
IGFL1	protein_coding
AC010332.1	unprocessed_pseudogene
NDUFV2P1	processed_pseudogene
RPL5P34	processed_pseudogene
MT-TQ	Mt_tRNA

Supplementary Table 4. Detailed information of the compounds, antibodies, viruses, and software used in this study.

REAGENT or RESOURCE	SOURCE	IDENTIFIER
**Antibodies against**		
IL-1β (D3U3E), rabbit	Cell Signaling Technology	12703
Cleaved IL-1β, rabbit	Cell Signaling Technology	83186S
NLRP3, rabbit	ThermoFisher Scientific	PA5-20838
OC43-N, mouse	Sigma-Aldrich	MAB9012
SRAS CoV 2-N, mouse	Thermo Fisher Scientific	MA5-29981
dsRNA, mouse	SCICONS	10010200
β-actin, mouse	Santa Cruz Biotechnology	sc-47778
Caspase 1, rabbit	Cell Signaling Technology	24232
Cleaved caspase 1, rabbit	Cell Signaling Technology	4199
NF κB, rabbit	Cell Signaling Technology	8242
Villin, mouse	Santa Cruz Biotechnology	sc-66022
Muc 2, mouse	Santa Cruz Biotechnology	sc-59859
CHGA, mouse	Santa Cruz Biotechnology	sc-393941
Epcam, rabbit	Abcam	ab71916
CD14-eFluor450	Thermo Fisher Scientific	11-0149-41
CD32-PE	Thermo Fisher Scientific	12-0329-42
CD68 Monoclonal Antibody (KP1)	Thermo Fisher Scientific	14-0688-82
HRP Horse Anti-Mouse IgG Detection Kit,	VectorLabs	MP-7402
CD16-FITC	Nuclilab	1F-399-T100
CD68-BV785	Biolegend	333826
CD80-PECy7	Biolegend	305218
HLA DR-BV605	Biolegend	307640
CD14-BV711	Biolegend	301838
CD45-APC-Fire750	Biolegend	304062
IgG1-FITC	BD Pharmingen	556026
IgG2b-BV785	Biolegend	402219
IgG1-PECy7	Biolegend	400126
IgG2a-BV605	Biolegend	400270
Phospho-STAT1 (Tyr701)	Cell Signalling Technology	7649
IRF-9 (D2T8M)	Cell Signalling Technology	76684
PKR (D7F7)	Cell Signalling Technology	12297
Phospho-eIF2α (Ser51)	Cell Signalling Technology	3398
IRDye® 680RD Goat anti-Mouse IgG (H + L)	Westburg BV	926-68070

IRDye® 800CW Goat anti-Rabbit IgG (H + L)	Westburg BV	926-32211
Alexa Fluor 488 Goat – anti-rabbit	Thermo Fisher Scientific	A32731
Alexa Fluor 555 Goat anti-Rabbit	ThermoFisher Scientific	A-21428
Alexa Fluor 594 Goat anti-Mouse	ThermoFisher Scientific	A32742
Alexa Fluor 647 Goat anti-Rabbit	Thermo Fisher Scientific	A-21245
**Virus strains**		
SARS-CoV-2 Omicron	Erasmus Medical Center	GenBank: MT270101
OC43	Erasmus Medical Center	GenBank: AY585228
Echovirus 1	Erasmus Medical Center	GenBank: AF029859
Echovirus 6	Erasmus Medical Center	GenBank: JQ929657
Rotavirus SA11	Erasmus Medical Center	GenBank: X16830
**Chemicals**		
Matrigel	Corning	356231
Immobilon® Block - FL (Fluorescent Blocker)	Merck Chemicals BV	WBAVDFL01
Intercept® (PBS) Blocking Buffer	LI-COR	927-70010
Immobilon-FL PVDF, 0.45 µm, 8.5 cm x 10 m roll	Merck Chemicals BV	IPFL85R
PrimeScript™ RT Master Mix (Perfect Real Time)	Takara Bio Europe S.A.S.	RR036A
Methanol	Boom	72,032,213.2500
TrypLE	ThermoFisher Scientific	12,604,013
HEPES	Lonza/Fisher Scientific	BE17-737E
N-2 supplement (50x)	Gibco/ThermoFisher Scientific	15,410,294
B27 supplement 50x without vitamin A	Gibco/ThermoFisher Scientific	12,587,001
Gastrin I	Sigma-Aldrich	G9145
n-Acetyl Cysteine	Sigma-Aldrich	A7250-5G
EGF	Peprotech	AF-100-15
A83-01	Cayman Chemical/Sanbio	9,001,799–25
Nicotinamide	Sigma-Aldrich	N0636-100G
SB 202190	Sigma-Aldrich	S7067-5MG
SB431542	Sigma-Aldrich	616464-5MG
Y-27632	MedChem Express/Bioconnect	HY-10583_10mg
DAPT Selleck	Chemicals/Bioconnect	S2215_10mg
Dexamethasone (Dex)	Sigma-Aldrich	D4902-100MG
6-mercaptopurine (6-MP)	MedChem Express	HY-13677
Methotrexate (MTX)	MedChem Express	HY-14519
Tofacitinib (Tof)	MedChem Express	HY-40354

Budesonide (Bud)	MedChem Express	HY-13580
5-aminosalicylic acid (5-ASA)	MedChem Express	HY-15027
Azathioprine (AZA)	MedChem Express	HY-B0256
Cyclosporin A (CSA)	Abcam	ab120114
Prednisolone (Pred)	Sigma-Aldrich	50-24-8
6-Thioguanine (6-TG)	Sigma-Aldrich	A4882-100MG
Sodium Acetate	Sigma-Aldrich	127-09-3
Sodium propionate	Sigma-Aldrich	137-40-6
Sodium butyrate	Sigma-Aldrich	156-54-7
Bay 11-7082	Sigma-Aldrich	19542-67-7
VX-765	Sigma-Aldrich	273404-37-8
MCC950	Sigma-Aldrich	256373-96-3
DMEM high glucose w/Na pyruvate w/ Stable glutamine	VWR International BV	L0193-500
PMA(12-O-Tetradecanoylphorbol 13-acetate)	Sigma-Aldrich Chemie BV	P1585
RPMI 1640 (STABLE GLUTAMINE)	Westburg BV	L0498-500
Iscove's MDM (-phenolred)	Life technologies	21056
F12 Nutrient Mixture (Ham)	Life technologies	31765
Poly vinyl alcoho	Sigma-Aldrich	P8136
Chemically Defined Lipids	Life technologies	11905031
Insulin-Transferrin-Selenium-X	Life technologies	51500
Monothioglycerol	Sigma-Aldrich	M6145-25mL
L-Ascorbic acid 2-phosphate	Sigma-Aldrich	A8960
Glutamax-1 supplement	Life technologies	35050
MEM non-essential amino acids	Life technologies	11140035
Accutase-Solution	PromoCell	C-41310
Human M-CSF	Miltenyi Biotec	130-096-492
RevitaCell Supplement	Life technologies	A26445-01
**Software**		
Adobe Illustrator CC	Adobe	https://www.adobe.com/products/illustrator.html
GraphPad Prism 8.0	GraphPad	Graphpad Software
ImageJ	NIH	https://ImageJ.nih.gov/ij/
Bedtools	University of Utah	https://github.com/arq5x/bedtools2
Integrative Genomics Viewer	Broad Institute of MIT and Harvard	https://software.broadinstitute.org/software/igv/

R	Rstudio	https://www.rstudio.com/products/ rstudio/download/
Fastp	OpenGene	https://github.com/OpenGene/fastp
Bowtie2	Johns Hopkins University	http://bowtie-bio.sourceforge.net/bowtie2/index.shtml
SAMtools	Broad Institute of MIT	http://www.htslib.org/download/
FlowJo	BD	https://www.flowjo.com/solutions/ flowjo/downloads

Supplementary Table 5. Primer sequences used in this study.

**Gene**	**Sequence (5’-3’)**
GAPDH-F	GTCTCCTCTGACTTCAACAGCG
GAPDH-R	ACCACCCTGTTGCTGTAGCCAA
IL-1β-F	CCACAGACCTTCCAGGAGAATG
IL-1β-R	GTGCAGTTCAGTGATCGTACAGG
IL-6-F	AGACAGCCACTCACCTCTTCAG
IL-6-R	TTCTGCCAGTGCCTCTTTGCTG
IL-8-F	GAGAGTGATTGAGAGTGGACCAC
IL-8-R	CACAACCCTCTGCACCCAGTTT
IL-10-F	TCTCCGAGATGCCTTCAGCAGA
IL-10-R	TCAGACAAGGCTTGGCAACCCA
IL-12-F	GACATTCTGCGTTCAGGTCCAG
IL-12-R	CATTTTTGCGGCAGATGACCGTG
IL-18-F	GATAGCCAGCCTAGAGGTATGG
IL-18-R	CCTTGATGTTATCAGGAGGATTCA
TNF α-F	CTCTTCTGCCTGCTGCACTTTG
TNF α-R	ATGGGCTACAGGCTTGTCACTC
GM-CSF-F	GGAGCATGTGAATGCCATCCAG
GM-CSF-R	CTGGAGGTCAAACATTTCTGAGAT
CCL-2-F	AGAATCACCAGCAGCAAGTGTCC
CCL-2-R	TCCTGAACCCACTTCTGCTTGG
CCL-4-F	GCTTCCTCGCAACTTTGTGGTAG
CCL-4-R	GGTCATACACGTACTCCTGGAC
CXCL-10-F	GGTGAGAAGAGATGTCTGAATCC
CXCL-10-R	GTCCATCCTTGGAAGCACTGCA
CXCL10-F	GGTGAGAAGAGATGTCTGAATCC
CXCL10-R	GAAGCACTGCA
OC43-F	AGCAACCAGGCTGATGTCAATACC
OC43-R	AGCAGACCTTCCTGAGCCTTCAAT
SARS-CoV-2-F	CAATGGTTTAACAGGCACAGG
SARS-CoV-2-R	CTCAAGTGTCTGTGGATCACG
EV-F	TCCTCCGGCCCCTGA
EV-R	RATTGTCACCATAAGCAGCCA
Rota-F	TGGTTAAACGCAGGATCGGA
Rota-R	AACCTTTCCGCGTCTGGTAG
IFN β-F	CTTGGATTCCTACAAAGAAGCAGC
IFN β-R	TCCTCCTTCTGGAACTGCTGCA
ISG15-F	CTCTGAGCATCCTGGTGAGGAA
ISG15-R	AAGGTCAGCCAGAACAGGTCGT
MX1-F	GGCTGTTTACCAGACTCCGACA
MX1-R	CACAAAGCCTGGCAGCTCTCTA
STAT1-F	ATGGCAGTCTGGCGGCTGAATT

STAT1-R	CCAAACCAGGCTGGCACAATTG
IRF1-F	GAGGAGGTGAAAGACCAGAGCA
IRF1-R	TAGCATCTCGGCTGGACTTCGA
IRF9-F	CCACCGAAGTTCCAGGTAACAC
IRF9-R	AGTCTGCTCCAGCAAGTATCGG
IFIT1-F	GCCTTGCTGAAGTGTGGAGGAA
IFIT1-R	ATCCAGGCGATAGGCAGAGATC
Villin-F	GCTGCTCTACACCTACCTCATC
Villin-R	TTCTGGTCCAGGATGACGGCTT
Muc-2 F	ACTCTCCACACCCAGCATCATC
Muc-2 R	GTGTCTCCGTATGTGCCGTTGT
hCHGA-F	TGACCTCAACGATGCATTTC
hCHGA-R	CTGTCCTGGCTCTTCTGCTC
