# Supplementary material for: Macrophage-augmented intestinal organoids model virus-host interactions in enteric viral diseases and facilitate therapeutic development
Source: Nat Commun. 2025 May 14;16:4475. doi: 10.1038/s41467-025-59639-9 (PMC12078800; doi:10.1038/s41467-025-59639-9)
Supplement: Supplementary file 3 — Description of Additional Supplementary Files [file 41467_2025_59639_MOESM3_ESM.pdf]

Description of Additional Supplementary Files

File Name: Supplementary Movie 1
Description: The morphology of first 18 hours after fragmented intestinal organoids assembling with CFSE-labelled macrophages, related to Fig.1B. Images were captured every 0.5 hours.

File Name: Supplementary Movie 2
Description: Raw fluorescent Z-stack images of MaugOs, related to Supplementary Fig.1I. Epithelial membrane of organoids was stained by EpCAM (red); THP-1 monocytes-derived macrophages was stained by CFSE (green), and cell nuclei was stained by DAPI (blue).
