# Supplementary material for: Macrophage-augmented intestinal organoids model virus-host interactions in enteric viral diseases and facilitate therapeutic development
Source: Nat Commun. 2025 May 14;16:4475. doi: 10.1038/s41467-025-59639-9 (PMC12078800; doi:10.1038/s41467-025-59639-9)
Supplement: Supplementary file 6 — Reporting Summary [file 41467_2025_59639_MOESM6_ESM.pdf]

nature portfolio

Corresponding author(s): Pengfei Li

Last updated by author(s): Apr 29, 2025

Reporting Summary

Nature Portfolio wishes to improve the reproducibility of the work that we publish. This form provides structure for consistency and transparency in reporting. For further information on Nature Portfolio policies, see our Editorial Policies and the Editorial Policy Checklist.

Statistics

For all statistical analyses, confirm that the following items are present in the figure legend, table legend, main text, or Methods section.

n/a	Confirmed	
☐	☒	The exact sample size (n) for each experimental group/condition, given as a discrete number and unit of measurement
☐	☒	A statement on whether measurements were taken from distinct samples or whether the same sample was measured repeatedly
☐	☒	The statistical test(s) used AND whether they are one- or two-sided<br>*Only common tests should be described solely by name; describe more complex techniques in the Methods section.*
☐	☒	A description of all covariates tested
☐	☒	A description of any assumptions or corrections, such as tests of normality and adjustment for multiple comparisons
☐	☒	A full description of the statistical parameters including central tendency (e.g. means) or other basic estimates (e.g. regression coefficient) AND variation (e.g. standard deviation) or associated estimates of uncertainty (e.g. confidence intervals)
☐	☒	For null hypothesis testing, the test statistic (e.g. F, t, r) with confidence intervals, effect sizes, degrees of freedom and P value noted<br>*Give P values as exact values whenever suitable.*
☒	☐	For Bayesian analysis, information on the choice of priors and Markov chain Monte Carlo settings
☒	☐	For hierarchical and complex designs, identification of the appropriate level for tests and full reporting of outcomes
☐	☒	Estimates of effect sizes (e.g. Cohen's d, Pearson's r), indicating how they were calculated

Our web collection on statistics for biologists contains articles on many of the points above.

Software and code

Policy information about availability of computer code

Data collection	ZEN Core (v3.6); LAS AF (v2); BD FACSDiva Software (v9.1); Odyssey 3.0 software (v5.2)
Data analysis	Prism (v8.0); Image J (v3.1); R (v3.6); FlowJo (v10.9); Bedtools (v2.31); Integrative Genomics Viewer (v2.1.2); Fastp (v0.23.2); Bowtie2 (v2.4.4); SAMtools (v0.1.19)

For manuscripts utilizing custom algorithms or software that are central to the research but not yet described in published literature, software must be made available to editors and reviewers. We strongly encourage code deposition in a community repository (e.g. GitHub). See the Nature Portfolio guidelines for submitting code & software for further information.

Data

Policy information about availability of data

All manuscripts must include a data availability statement. This statement should provide the following information, where applicable:

- Accession codes, unique identifiers, or web links for publicly available datasets
- A description of any restrictions on data availability
- For clinical datasets or third party data, please ensure that the statement adheres to our policy

Data that support the findings of this study are available in the main text, supplementary information and source data. RNA sequencing data are publicly available at the NCBI database under accession code GSE295767.

Research involving human participants, their data, or biological material

Policy information about studies with human participants or human data. See also policy information about sex, gender (identity/presentation), and sexual orientation and race, ethnicity and racism.

Reporting on sex and gender	N/A
Reporting on race, ethnicity, or other socially relevant groupings	N/A
Population characteristics	N/A
Recruitment	N/A
Ethics oversight	N/A

Note that full information on the approval of the study protocol must also be provided in the manuscript.

Field-specific reporting

Please select the one below that is the best fit for your research. If you are not sure, read the appropriate sections before making your selection.

☒ Life sciences ☐ Behavioural & social sciences ☐ Ecological, evolutionary & environmental sciences

For a reference copy of the document with all sections, see nature.com/documents/nr-reporting-summary-flat.pdf

Life sciences study design

All studies must disclose on these points even when the disclosure is negative.

Sample size	Sample sizes were determined based on prior experience and published literature, with details provided in the Figure Legends.
Data exclusions	No data exclusion
Replication	All experimental replicates were reported in figure legends and performed at least twice to show reproducible patterns. The results were generated from multiple organoids lines and different types of macrophages, as well as five viruses. Well-planned replications were all successful.
Randomization	MaugOs were randomly selected for groups of infection, or (drug) treatment.
Blinding	Official blinding protocols were not part of this in vitro study due to its experimental framework.

Reporting for specific materials, systems and methods

We require information from authors about some types of materials, experimental systems and methods used in many studies. Here, indicate whether each material, system or method listed is relevant to your study. If you are not sure if a list item applies to your research, read the appropriate section before selecting a response.

Materials & experimental systems

n/a	Involved in the study	
☐	☒	Antibodies
☐	☒	Eukaryotic cell lines
☒	☐	Palaeontology and archaeology
☒	☐	Animals and other organisms
☒	☐	Clinical data
☒	☐	Dual use research of concern
☒	☐	Plants

Methods

n/a	Involved in the study	
☒	☐	ChIP-seq
☐	☒	Flow cytometry
☒	☐	MRI-based neuroimaging

Antibodies

Antibodies used	For western blot:<br>IL-1β (D3U3E) (Cell Signaling Technology, 12703, 1:1000)

Cleaved IL-1β (Cell Signaling Technology, 83186, 1:1000)
NLRP3 (Thermo Fisher Scientific, PA5-20838, , 1:1000)
β-actin (Santa Cruz Biotechnology, sc-47778, , 1:1000)
Caspase 1 (Cell Signaling Technology, 24232, , 1:1000)
Cleaved caspase 1 (Cell Signaling Technology, 4199, 1:1000)
NF-κB (Cell Signaling Technology, 8242, 1:1000)
Phospho-STAT1 (Tyr701) (Cell Signaling Technology, 7649, 1:1000)
IRF-9 (D2T8M) (Cell Signaling Technology, 76684, 1:1000)
PKR (D7F7) (Cell Signaling Technology, 12297, 1:1000)
Phospho-eIF2α (Ser51) (Cell Signaling Technology, 3398, 1:1000)
IRDye® 680RD Goat anti-Mouse IgG (H+L) (Westburg BV, 926-68070, 1:5000)
IRDye® 800CW Goat anti-Rabbit IgG (H+L) (Westburg BV, 926-32211, 1:5000)
For Immunostaining:
OC43-N (Sigma-Aldrich, MAB9012, 1:500)
SARS-CoV-2-N (Thermo Fisher Scientific, MA5-29981, 1:500)
dsRNA (SCICONS, 10010200, 1:500)
Villin (Santa Cruz Biotechnology, sc-66022, 1:100)
Muc2 (Santa Cruz Biotechnology, sc-59859, 1:100)
CHGA (Santa Cruz Biotechnology, sc-393941, 1:100)
Epcam (Abcam, ab71916, 1:500)
Alexa Fluor 488 Goat anti-Rabbit (Thermo Fisher Scientific, A32731, 1:1000)
Alexa Fluor 555 Goat anti-Rabbit (Thermo Fisher Scientific, A-21428, 1:1000)
Alexa Fluor 594 Goat anti-Mouse (Thermo Fisher Scientific, A32742, 1:1000)
Alexa Fluor 647 Goat anti-Rabbit (Thermo Fisher Scientific, A-21245, 1:1000)
For Flow Cytometry anlysis:
CD14-eFluor450 (Thermo Fisher Scientific, 11-0149-41, 1:50)
CD32-PE (Thermo Fisher Scientific, 12-0329-42, 1:200)
CD68 (KP1) (Thermo Fisher Scientific, 14-0688-82, 1:200)
CD16-FITC (Nuclilab, 1F-399-T100, 1:50)
CD68-BV785 (BioLegend, 333826, 1:200)
CD80-PECy7 (BioLegend, 305218, 1:25)
HLA-DR-BV605 (BioLegend, 307640, 1:200)
CD14-BV711 (BioLegend, 301838, 1:50)
CD45-APC-Fire750 (BioLegend, 304062, 1:30)
IgG1-FITC (BD Pharmingen, 556026, 1:20)
IgG2b-BV785 (BioLegend, 402219, 1:320)
IgG1-PECy7 (BioLegend, 400126, 1:25)
IgG2a-BV605 (BioLegend, 400270, 1:400)

Validation

The antibodies selected were validated by previous studies from our own research groups or from the research field, as well as verification statement on producer website.

Eukaryotic cell lines

Policy information about cell lines and Sex and Gender in Research

Cell line source(s)

Cell lines (including A549, Huh7, Vero-E6, MA-104, THP-1) were stocked in our lab and originally from the American Type Culture Collection.

Authentication

Cell lines in this study were analyzed by genotyping

Mycoplasma contamination

Cell lines were routinely confirmed by commercial company to be mycoplasma negative.

Commonly misidentified lines
(See ICLAC register)

No commonly misidentified cell lines were used in the study.

Plants

Seed stocks

N/A

Novel plant genotypes

N/A

Authentication

N/A

Flow Cytometry

Plots

Confirm that:

☒ The axis labels state the marker and fluorochrome used (e.g. CD4-FITC).

☒ The axis scales are clearly visible. Include numbers along axes only for bottom left plot of group (a 'group' is an analysis of identical markers).

☒ All plots are contour plots with outliers or pseudocolor plots.

☒ A numerical value for number of cells or percentage (with statistics) is provided.

Methodology

Sample preparation	Macrophage cell lines with or without differentiation were collected for staining cell surface markers
Instrument	BD FACSCanto
Software	Flowjo
Cell population abundance	The target cell population is highly abundant. No cell populations were sorted.
Gating strategy	Macrophages in this study were analyzed using standard gating strategy to just confirm the phenotype. See details in Supplementary Figure 1

☒ Tick this box to confirm that a figure exemplifying the gating strategy is provided in the Supplementary Information.
